# Racial differences in Urinary Bladder Cancer in the United States

**DOI:** 10.1038/s41598-018-29987-2

**Published:** 2018-08-21

**Authors:** Yu Wang, Qian Chang, Yang Li

**Affiliations:** 1 Center for Applied Statistics, Renmin University of China, Haidian Qu, China; 2 School of Statistics, Renmin University of China, Haidian Qu, China

## Abstract

Urinary bladder cancer (UBC) has a high incidence rates in many southern and eastern European countries, in parts of Africa and the Middle East, and in North America. It exhibits a wide variety of histological types that goes from less aggressive to rapid-growing ones. In order to compare the different presentations, etiologies, and prognoses among racial groups, including NHW (non-Hispanic white), HW (Hispanic white), blacks, and API (Asian and Pacific Islander), we analyzed the UBC patients diagnosed between 1973 and 2014 using SEER (Surveillance, Epidemiology, and End Results) database. Patient characteristics, age-adjusted incidence rates, and survival were compared across races. There are significant racial differences in patients’ characteristics, including gender, marital status, age at diagnosis, treatment strategies, grade, stage, survival time, and so on. Overall, non-Hispanic whites have the highest incidence rate, followed by blacks, Hispanic whites, and APIs. In the analysis of survival, significant racial differences exist when stratified by gender, age group, histological type, stage, location and treatment strategies. Racial differences exist among UBC patients in the United States in terms of characteristics, incidence, and survival. Future studies may collect and analyze more data for comprehensive description and interpretation of the racial differences.

## Introduction

Study on racial differences among cancer patients have attracted extensive attention^1–3^. It can assist diagnosis, implementation of tailored treatment strategies, and elimination of racial disparity. However, the related research on urinary bladder cancer (UBC) remains limited. Urinary bladder cancer is a type of cancer that arises from the epithelial lining of the urinary bladder. UBC has the fifth highest incidence among all cancers in the United States; an estimated 76,960 new cases of UBC and 16,390 deaths from UBC occurred in 2016^4^. Incidence rates of UBC are high in many southern and eastern European countries, in parts of Africa and the Middle East, and in North America^5^. UBC has many histological types. Transitional Cell Carcinoma (TCC) is the most common histological type of primary UBC, which comprises more than 90% of all bladder tumors^6^. Except for TCC, this article also considers two other histological types which are squamous cell carcinoma (SCC) and Carcinoma. TCC, the most common bladder cancer, arises from the transitional epithelium and the pattern of growth of TCC can be papillary, sessile, or carcinoma-*in-situ* (CIS)^7^. So, TCC can be divided into two types: PTCC (Papillary Transitional Cell Carcinoma) and NPTCC (Non-Papillary Transitional Cell Carcinoma). In the type of TCC, PTCC accounts for a large proportion, and it’s nearly 70%. PTCC are often multifocal and only occasionally progress^8^. Compared with NPTCC, PTCC has a higher differentiated degree, so its malignant degree is lower and the survival rate of patients is usually higher. SCC (Squamous Cell Carcinoma) is an epithelial malignant tumor originating from the bladder mucosa^8^. The incidence rate of bladder squamous cell carcinoma in bladder malignancies worldwide varies widely. It is usually associated with the chronic urinary infection caused by schistosoma haematobium (also known as schistosomiasis or bilharzia). In regions where this infection is endemic, like Egypt, SCC was for years the dominant histological type of UBC^9^. Carcinoma Malignant epithelial tumor is another type of malignant tumor derived from bladder epithelial tissue. It is a rare histological type too and with a very scarce literature to pay attention to it. Urinary bladder cancer is the second most common genitourinary malignant disease in the United States and the 13^th^ most common cause of death worldwide. In western countries, the incidence rate of UBC continues to rise in recent years^5^. There are a lot of risk factors for UBC. Smoking is recognized as the most important risk factor for UBC and is estimated to account for 50% of tumors. Occupational exposure to carcinogens— namely, aromatic amines (benzidine, 4-aminobiphenyl, 2-naphthylamine, 4-chloro-o-toluidine), polycyclic aromatic hydrocarbons, and chlorinated hydrocarbons—is viewed as the second most important risk factor^10^. Some other risk factors just like coffee drinking, artificial sweeteners, and nutrition intake were discussed by some other researchers^11,12]^. Treatment options for bladder cancer depend on a number of factors, including the type of cancer, the grade, and the stage and so on. The most commonly used treatment strategy is transurethral resection of the bladder tumor. Other treatments include the cystectomy, the intravesical chemotherapy, the systemic chemotherapy, the radiation therapy, and the immunotherapy^13^. Cystectomy has the best treatment effect. It can prevent recurrence to a large extent. Transurethral resection of the bladder tumor followed by combined chemotherapy and radiation therapy also can achieve as effective as cystectomy^14^. Although the five-year survival rate of UBC is good (62–88%), the recurrence rate is also high^15^. There are a lot of prognostic factors for recurrence of UBC, including the number of tumors present at diagnosis, the recurrence rate in the previous period, the size of tumor, and the grade of tumor^16,17^.

This study aims to comprehensively describe the racial differences among UBC patients in the United States in terms of patients’ characteristics, clinical-pathologic features, incidence rates, and survival rates by analyzing the SEER database. Recently, researches on racial differences among cancer patients have attracted extensive attention^1–3^, but the discussion on UBC remains scarce. The available UBC studies may have limitations by either the numbered racial groups or the specified outcomes. For instance, Scosyrev and others^18^ only compared blacks and whites. They found that African American patients present with less favorable tumor characteristics compared with white patients, accounting for a much higher UBC mortality experienced by these groups. Burger and others^10^ (2013) mainly studied on risk factors of urothelial bladder cancer. Although the racial difference of UBC are mentioned in his article, the discussion only focuses on survival outcome as well as the discussion about racial difference of UBC is not deep enough. Yee and others^19^ also just focus on survival outcome for different racial groups in UBC. They found that ethnic disparities in bladder cancer survival persist between whites and blacks, whereas survival in other ethnic minority groups appears similar to that of whites. However, our study on UBC are more comprehensive than previous study. We compare a wider spectrum of racial groups, including NHW (non-Hispanic white), HW (Hispanic white), blacks, and API (Asian and Pacific Islander), and analyze multiple aspects, including patients’ characteristics, clinical-pathological features, incidence rate, and survival rate.

## Methods

### Source population

The Surveillance, Epidemiology, and End Results (SEER, http://seer.cancer.gov) database of the National Cancer Institute is utilized to provide population-based sample data in this study. This program collects information on cancer statistics in an effort to reduce the cancer burden among the U.S. population. Approximately 9.5%, 14%, and 28% of the U.S. population are analyzed by covering registry 9, 13, and 18 respectively, and the International Classification of Disease for Oncology (ICD-O-3) site code C670- C679 are referred to identify UBC cases in those population. Tumor sites and histological types are coded based on WHO specified criteria in ICD-O-3. And histological types are also grouped by ICD-O-3. Patients with PTCC, NPTCC, Carcinoma, and SCC are identified by the histology codes 8130–8131, 8120–8124, 8010–8015, and 8070–8078, respectively. The division of treatment variable is classified comprehensively in this study. There are three main treatment variables in the SEER database, including surgery, radiotherapy, and chemotherapy. We consider a variety of combinations of these three methods including surgery, chemotherapy, radiation, SR (surgery and radiation), SC (surgery and chemotherapy), RC (radiation and chemotherapy), SRC (surgery, radiation and chemotherapy) and no treatment.

Different SEER registry groupings are used to maximize the sample size. In the analysis of patients’ characteristics and clinical-pathological features, SEER 9 data is applied and the following information is included: cancers diagnosed between 1973 and 2014, gender, marital status, age at diagnosis, age group (0–39 years, 40–64 years, 65–84 years, 85+ years), survival time, histological types, grade, stage (II–IV), treatment, tumor size, lymph node site (nodal, extra-nodal), and location. SEER 13 data containing detailed race and incidence information for cancers diagnosed between 1992 and 2014, is used in the analysis of incidence rate. Cancer diagnosed between 1973 and 2008 with follow-ups up to the end of 2013 from SEER 18 data is analyzed in the survival analysis.

### Statistical analysis

This study uses Chi-square tests and ANOVA analysis in order to compare the patients’ characteristics and clinical pathologic features across racial groups by SAS version 9.4. The age-standardized incidence rates and five-year relative survival rates are calculated by SEER* Stat software using US 2000 Census data. Multivariate Cox regressions are also performed when adjusting for gender, age, histological type, stage, location and treatment strategy.

### Data availability

The data is available on the Surveillance, Epidemiology, and End Results (SEER, http://seer.cancer.gov) database.

## Result

### Patients’ characteristics and clinical-pathological features

Results are presented in Table 1. There are overall more male patients. The gender distributions are different across races (p-value<0.001), with NHWs having the most male patients (75.5%) and blacks having the least (66.5%). Most UBC patients are married (65.0%). Different racial groups have significantly different marital status (p-value<0.001). Among API patients, 70.2% are married. In contrast, only 45.8% of blacks are married. The age at diagnosis are also significantly different across races (p-value <0.001). Non-Hispanic whites have the highest age at diagnosis (70.9 years) and Hispanic whites have the lowest age at diagnosis (66.6 years).The median survival time for all patients is 50.0 months. Non-Hispanic whites have the longest survival time with a median of 51 months, and blacks have the shortest survival time with a median of 33 months. The distribution of histological types has racial differences (p-value <0.001) too. Non-Hispanic whites have most PTCC (66.3%), whereas blacks have most NPTCC (27.6%), CARCINOMA (1.6%), and SCC (4.1%). The tumor grade is different across different races (p-value<0.001). APIs (27.9%) have more grade IV patients, blacks (31.2%) have more grade III patients, whereas NHWs (34.5%) and HWs (33.3%) have more grade II patients. Most UBCs are diagnosed at an early stage. In our analysis, 76.4% tumors are stage II. Non-Hispanic whites have most tumors at stage II (77.2%), blacks have most stage III (26.6%) and stage IV (7.8%). For all patients, the average tumor size is 41.1mm. Blacks have the largest tumor size (46.3mm) while APIs have the smallest tumor size (38.5mm). NHWs (92.5%) have the highest rates of all nodes negative while blacks have the lowest (90.1%). This racial difference is significant. Further, there is a significant racial difference in tumor location distribution (p-value <0.001). The most common tumor location is the lateral wall of bladder. Non-Hispanic whites have the highest rates of lateral wall of bladder, trigone of bladder, ureteric orifice (20.0%, 6.0%, 4.8%, respectively), blacks have the highest rates of overlapping lesion of bladder, dome of bladder, anterior wall of bladder (12.0%, 4.7%, 2.3%, respectively) and APIs have the highest rates of posterior wall of bladder, bladder neck, urachus (9.4%, 3.8%, 0.4%, respectively). Different races also have different treatment strategies (p-value <0.001). For all patients, the surgery and SC account for more than 80%. Non-Hispanic whites have the highest rates of surgery (77.6%), APIs have the highest rates of surgery and chemotherapy (14.1%), whereas blacks have the highest rates of no treatment (9.7%), SR (4.6%), SRC (3.1%), chemotherapy (1.0%), radiation (1.0%) and RC (0.3%), respectively.

**Table 1:** UBC patients’ characteristics and clinical-pathologic features for the whole cohort and different racial groups.

	Total (n = 377400)	NHW (n = 325612)	HW (n = 17613)	Black (n = 19646)	API (n = 14147)	P
Gender						<0.001
Male	282714 (74.9)	245806 (75.5)	12953 (73.5)	13073 (66.5)	10586 (74.8)	
Female	94686 (25.1)	79806 (24.5)	4660 (26.5)	6573 (33.5)	3561 (25.2)	
Marital Status						<0.001
Single	34171 (9.6)	26861 (8.7)	2237 (13.4)	3759 (20.4)	1258 (9.4)	
Married	231638 (65.0)	203205 (66.1)	10354 (62.1)	8452 (45.8)	9409 (70.2)	
Separated/d/w	90451 (25.4)	77329 (25.2)	4078 (24.5)	6251 (33.9)	2736 (20.4)	
Age group						
0–39	5124 (1.4)	3970 (1.2)	521 (3.0)	364 (1.9)	246 (1.7)	<0.001
40–64	103273 (27.4)	87161 (26.8)	5533 (31.4)	6550 (33.3)	3883 (27.5)	
65–84	223595 (59.3)	194596 (59.8)	9714 (55.2)	10850 (55.2)	8248 (58.3)	
85+	45398 (12.0)	39881 (12.3)	1844 (9.6)	1880 (9.6)	1767 (12.5)	
Age at diagnosis	70.7 ± 14.9	70.9 ± 12.4	66.6±13.7	68.6 ± 12.9	70.7 ± 18.6	<0.001
Survival time (month)	50.0 ± 93.0	51.0 ± 95.0	43.0 ± 85.0	33.0 ± 77.0	48.0 ± 94.0	<0.001
Histological types						<0.001
PTCC	246724 (65.4)	215800 (66.3)	11130 (63.2)	10786 (54.9)	8721 (61.7)	
NPTCC	102611 (27.2)	87054 (26.7)	4862 (27.6)	6362 (32.4)	4271 (30.2)	
CARCINOMA	6364 (1.7)	5303 (1.6)	311 (1.8)	476 (2.4)	270 (1.9)	
SCC	5194 (1.4)	4091 (1.3)	303 (1.7)	630 (3.2)	166 (1.2)	
Others	16507 (4.4)	13364 (4.1)	1007 (5.7)	1392 (7.1)	719 (5.1)	
Grade						<0.001
Grade I	53673 (16.9)	47393 (17.3)	2344 (16.1)	2344 (14.7)	1541 (13.1)	
Grade II	107825 (34.0)	94567 (34.5)	4851 (33.3)	4537 (28.4)	3769 (31.9)	
Grade III	84665 (26.7)	72609 (26.5)	3806 (26.1)	4975 (31.2)	3206 (27.1)	
Grade IV	70885 (22.4)	59857 (21.8)	3575 (24.5)	4101 (25.7)	3295 (27.9)	
Stage						<0.001
stage II	275889 (76.4)	241037 (77.2)	12403 (74.0)	12073 (65.6)	10075 (74.6)	
stage III	70845 (19.6)	59712 (19.1)	3414 (20.4)	4885 (26.6)	2791 (20.7)	
stage IV	14422 (4.0)	11401 (3.7)	936 (5.6)	1430 (7.8)	645 (4.8)	
Tumor size (mm)	41.1 ± 49.2	40.9 ± 47.2	41.3 ± 61.1	46.3 ± 58.9	38.5 ± 58.2	<0.001
Regional nodes						<0.001
All nodes negative	54839 (92.2)	47552 (92.5)	2518 (90.7)	2763 (90.1)	1978 (90.7)	
≥1 nodes positive	4642 (7.8)	3873 (7.5)	259 (9.3)	305 (10.0)	204 (9.4)	
Location						<0.001
NOS	156540 (41.5)	134286 (41.2)	7325 (41.6)	8882 (45.2)	5861 (41.4)	
Lateral wall of bladder	74042 (19.6)	65004 (20.0)	3266 (18.5)	3061 (15.6)	2640 (18.7)	
Overlapping lesion of bladder	41538 (11.0)	35805 (11.0)	1936 (11.0)	2361 (12.0)	1409 (10.0)	
Posterior wall of bladder	32095 (8.5)	27599 (8.5)	1632 (9.2)	1515 (7.7)	1328 (9.4)	
Trigone of bladder	22677 (6.0)	19673 (6.0)	1059 (6.0)	1100 (5.6)	830 (5.9)	
Ureteric orifice	17362 (4.6)	15538 (4.8)	685 (3.9)	560 (2.8)	555 (3.9)	
Dome of bladder	13755 (3.6)	11483 (3.5)	720 (4.1)	930 (4.7)	611 (4.3)	
Bladder neck	11734 (3.1)	9878 (3.0)	573 (3.3)	729 (3.7)	543 (3.8)	
Anterior wall of bladder	7116 (1.9)	5978 (1.8)	366 (2.1)	453 (2.3)	313 (2.2)	
Urachus	541 (0.1)	368 (0.1)	60 (0.3)	55 (0.3)	57 (0.4)	
Treatment						<0.001
Surgery	285607 (76.9)	248900 (77.6)	13244 (76.1)	13248 (69.0)	10215 (73.2)	
SC	41615 (11.2)	35417 (11.0)	1929 (11.0)	2308 (12.0)	1961 (14.1)	
No treatment	20298 (5.5)	16480 (5.1)	1241 (7.1)	1741 (9.7)	836 (6.0)	
SR	11555 (3.1)	9822 (3.1)	405 (2.3)	883 (4.6)	445 (3.2)	
SRC	7905 (2.1)	6651 (2.1)	349 (2.0)	589 (3.1)	316 (2.3)	
Chemotherapy	2017 (0.5)	1619 (0.5)	137 (0.8)	186 (1.0)	75 (0.5)	
Radiation	1706 (0.5)	1380 (0.4)	65 (0.4)	183 (1.0)	78 (0.6)	
RC	562 (0.2)	428 (0.1)	34 (0.2)	66 (0.3)	34 (0.2)	

Cancers diagnosed 1973–2014 in the SEER 9 database. For a survival time, median ± interquartile range, for age at diagnosis and tumor size, mean ± standard deviation and for a categorical variable, count (percentage).

### Incidence rates

The overall age-adjusted incidence rate is 19.53 per 100,000 person-years (Table 2). Incidence rates increase with age. The four age groups have incidence rates 0.43, 15.36, 110.28, 154.23, respectively. Overall, non-Hispanic whites have the highest incidence rate, and the incidence rate is far greater than those of the other races. For the four age groups, non-Hispanic whites have the highest incidence rate, followed by blacks. APIs have the lowest rate. Overall, male have much higher incidence rate (34.46) than female (8.62). For both male and female, NHWs have the highest incidence rate, followed by blacks and then HWs. For the four major histological types, PTCC has the highest incidence rate, followed by NPTCC and then carcinoma. For PTCC and NPTCC, NHWs have the highest incidence rate (16.14 and 5.59, respectively), followed by blacks. While for Carcinoma, non-Hispanic whites and blacks have the same highest incidence rate (0.32). For SCC, blacks have the highest incidence rate (0.33), followed by NHWs (0.25). For all the four major histological types, APIs have the lowest incidence rate.

**Table 2:** Age-adjusted UBC incidence rates per 100,000 person-years for the whole cohort and different racial groups, stratified by gender, age and histological type.

	**Total**	**NHW**	**HW**	**Black**	**API**
**All ages**	19.53 (19.44–19.62)	23.09 (22.97–23.21)	11.10 (10.86–11.34)	12.60 (12.33–12.87)	9.51 (9.31–9.71)
0–39	0.43 (0.41–0.44)	0.57 (0.55–0.60)	0.25 (0.22–0.28)	0.27 (0.23–0.31)	0.18 (0.15–0.22)
40–64	15.36 (15.22–15.50)	19.11 (18.92–19.31)	7.87 (7.59–8.17)	10.22 (9.85–10.59)	6.48 (6.21–6.75)
65–84	110.28 (109.60–110.97)	129.22 (128.34–130.10)	62.72 (60.97–64.52)	69.70 (67.74–71.70)	53.64 (52.19–55.12)
≥85	154.23 (152.15–156.34)	169.34 (166.86–171.85)	103.75 (97.18–110.66)	104.18 (97.49–111.20)	95.93 (90.51–101.59)
**Gender**					
Male	34.46 (34.26–34.65)	40.58 (40.33–40.83)	19.41 (18.90–19.92)	20.78 (20.22–21.35)	16.57 (16.16–16.98)
Female	8.62 (8.53–8.70)	10.00 (9.90–10.11)	5.25 (5.03–5.46)	7.25 (6.99–7.52)	4.24 (4.06–4.42)
**Histological types**					
PTCC	13.37 (13.29–13.45)	16.14 (16.04–16.24)	7.08 (6.89–7.27)	7.23 (7.03–7.44)	6.06 (5.90–6.22)
NPTCC	4.91 (4.86–4.96)	5.59 (5.53–5.65)	3.07 (2.95–3.20)	3.97 (3.82–4.12)	2.82 (2.71–2.93)
CARCINOMA	0.23 (0.22–0.24)	0.32 (0.31–0.34)	0.21 (0.18–0.24)	0.32 (0.28–0.36)	0.17 (0.14–0.20)
SCC	0.30 (0.28–0.31)	0.25 (0.24–0.26)	0.19 (0.16–0.22)	0.33 (0.29–0.38)	0.07 (0.06–0.09)

Diagnoses in the period of 1992–2014 in the SEER 13 database. In each cell, estimated rate (95% confidence interval). Rates were age-adjusted using the U.S. 2000 Census population.

### Survival rates

**Figure 1:**
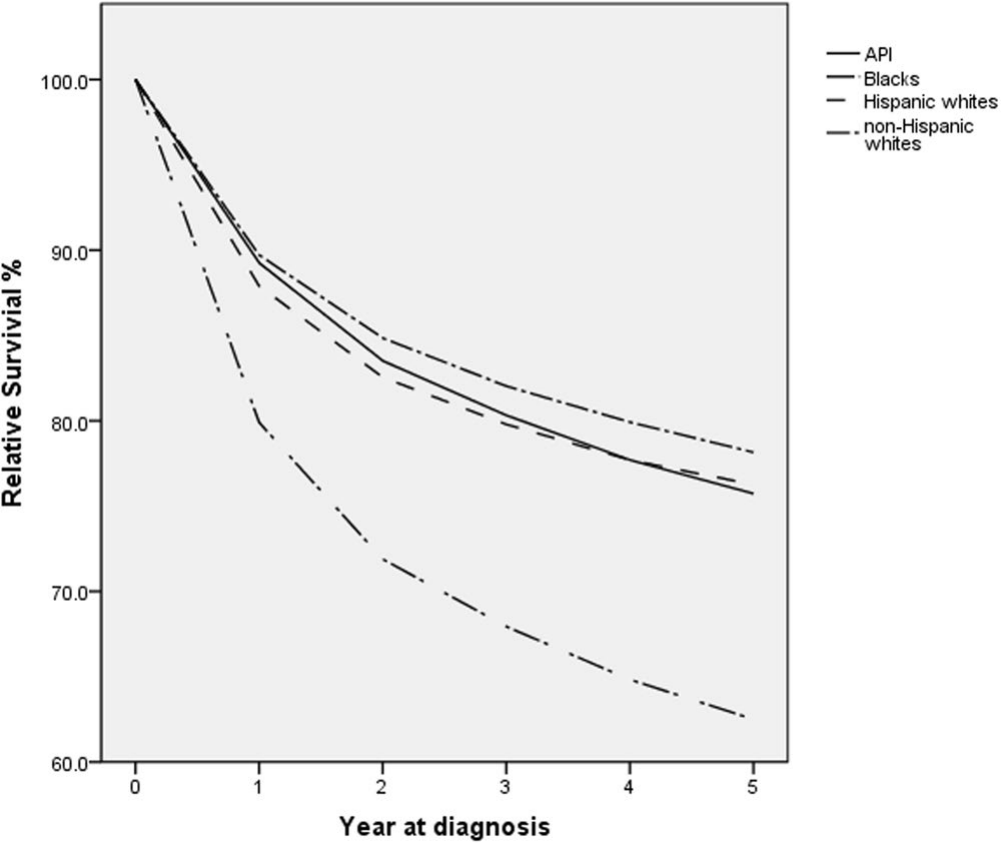
Relative survival up to five years for different racial groups. Cancers diagnosed in the period of 1973–2008, and followed up to 12/31/2013.

Figure 1 shows the unadjusted survival rate for five years. Non-Hispanic whites have better survival rates at all times, whereas the survival curves of other racial groups may cross. Blacks have a much lower survival rate than other racial groups. The five-year relative survival rates, stratified by gender, age group, histological types, stage, location, and treatment strategies are shown in Table 3. When stratified by gender, for male or female, non-Hispanic whites have the best survival rate and blacks have the worst. The racial differences are significant in multivariate Cox regression. When stratified by age, for the <40, 65–84 and 85+ years group, non-Hispanic whites all have the best survival rate and blacks all have the worst. For 40–64 years’ group, both non-Hispanic whites and APIs have the best survival rate and blacks still have the worst. The racial differences are significant in multivariate analysis. When stratified by histological type, for PTCC, both non-Hispanic whites and Hispanic whites have the best five-year survival rate (89.0%), followed by APIs (86.2%) and blacks (80.7%). For NPTCC, APIs have the best survival rate (61.3%), followed by NHWs (58.1%), HWs (56.1%) and blacks (42.8%). For Carcinoma, Hispanic whites have the best survival rate (50.2%), followed by NHWs (48.0%), APIs (37.9%) and blacks (35.1%). For SCC, APIs have the best survival rate (32.2%), followed by non-Hispanic white (28.0%), blacks (20.2%) and Hispanic whites (18.5%). For these histological types, the multivariate Cox regression suggests significant racial differences. When stratified by stage, for stage II, Hispanic whites have the best five-year survival rate (91.1%), followed by non-Hispanic whites (90.9%), APIs (89.0%) and blacks (83.6%). For stage III, APIs have the best survival rate (47.0%), followed by Hispanic whites (44.4%), non-Hispanic whites (43.4%) and blacks (33.8%). For stage IV, Hispanic whites have the best survival rate (7.0%), followed by non-Hispanic whites (5.9%), blacks (5.6%) and APIs (5.1%). For all the three tumor stages, the racial differences are significant in multivariate Cox regression. When stratified by tumor location, for trigone of the bladder, both non-Hispanic whites and Hispanic whites have the best survival rate (78.2%) and blacks have the worst (62.6%). For lateral wall of bladder, anterior wall of bladder, bladder neck, Ureteric orifice, and NOS, non-Hispanic whites have the best survival rate (85.9%, 72.3%, 75.3%, 56.3%, 76.2%, respectively) and blacks have the worst (72.9%, 56.7%, 56.2%, 39.6%, 59.2%, respectively). For dome of bladder and posterior wall of bladder, Hispanic whites have the best survival rate, and blacks still have the worst (64.1%, 70.8%). For ureteric orifice, non-Hispanic whites have the best survival rate (90.4%), and APIs have the worst (84.6%). For overlapping lesion of bladder, APIs have the highest five-year survival rate (65.8%) and blacks have the worst (52.2%). The racial differences are significant in a multivariate Cox regression for all locations except urachus. When stratified for treatment strategies, for surgery, non-Hispanic whites have the best survival rate (39.2%) while blacks have the worst (25.4%). For the SRC and SR, APIs have the best survival rate (40.2%, 36.8%, respectively) and blacks have the worst (26.9%, 22.3%, respectively). For these three treatment strategies, the racial differences are significant in a multivariate Cox regression. For other treatment strategies, there is no significant racial difference from the results of multivariate Cox regression.

**Table 3:** Survival rates of UBC for different racial groups, stratified by gender, age, histologic type, stage at diagnosis, location and treatment.

**Survival rates**	**Total**	**NHW**	**HW**	**Black**	**API**	**P**
**Gender**	77.4 (77.1–77.6)	78.2 (77.9–78.4)	76.3 (75.1–77.3)	62.5 (61.4–63.6)	75.7 (74.5–76.9)	
Male	79.0 (78.7–79.3)	79.5 (79.2–79.8)	78.9 (77.5–80.1)	67.5 (66.1–68.9)	77.5 (76.2–78.8)	<0.001
Female	72.5 (72.0–73.0)	74.1 (73.6–74.6)	69.01 (66.9–71.0)	53.1 (51.2–54.9)	70.2 (67.8–72.5)	<0.001
**Age group**						
0–39	92.7 (91.9–93.5)	94.0 (93.1–94.8)	89.3 (85.6–92.1)	83.0 (78.1–86.9)	89.4 (83.5–93.2)	0.001
40–64	84.1 (83.8–84.4)	85.1 (84.8–85.4)	81.4 (79.9–82.7)	69.0 (67.4–70.5)	85.1 (83.4–86.5)	<0.001
64–84	75.5 (75.2–75.8)	76.3 (76.0–76.7)	73.9 (72.2–75.4)	59.3 (57.6–60.9)	74.3 (72.6–75.8)	<0.001
85+	60.7 (59.3–62.0)	61.5 (60.0–63.0)	60.5 (53.8–66.6)	45.0 (39.4–50.5)	52.0 (46.4–57.3)	<0.001
**Histologic type**						
PTCC	88.7 (88.4–89.0)	89.0 (88.7–89.3)	89.0 (87.6–90.1)	80.7 (79.2–82.1)	86.2 (84.8–87.5)	<0.001
NPTCC	57.4 (56.9–57.9)	58.1 (57.6–58.7)	56.1 (53.9–58.3)	42.8 (41.0–44.6)	61.3 (59.0–63.5)	<0.001
Carcinoma	47.0 (45.0–48.9)	48.0 (45.8–50.1)	50.2 (41.5–58.3)	35.1 (287–41.6)	37.9 (29.4–46.3)	0.005
SCC	26.6 (25.0–28.3)	28.0 (26.1–29.9)	18.5 (12.9–25.1)	20.2 (16.1–24.7)	32.2 (22.1–42.7)	0.003
**Stage at diagnosis**						
Stage II	90.7 (90.4–90.9)	90.9 (90.6–91.2)	91.1 (89.9–92.2)	83.6 (82.2–84.9)	89.0 (87.7–90.2)	<0.001
Stage III	42.9 (42.4–43.5)	43.4 (42.8–44.0)	44.4 (41.9–46.9)	33.8 (31.9–35.7)	47.0 (44.3–49.7)	<0.001
Stage IV	5.9 (5.4–6.5)	5.9 (5.3–6.5)	7.0 (4.9–9.6)	5.6 (4.1–7.4)	5.1 (3.0–8.0)	0.024
**Location**						
Trigone of bladder	77.6 (76.6–78.5)	78.2 (77.2–79.3)	78.2 (73.5–82.1)	62.6 (57.8-67.0)	77.6 (72.6–81.8)	<0.001
Dome of bladder	71.6 (70.2–72.9)	71.6 (70.1–73.0)	77.3 (71.107–82.4)	64.1 (58.8–68.9)	75.1 (69.0–80.2)	<0.001
Lateral wall of bladder	85.4 (84.9–85.9)	85.9 (85.4–86.4)	85.6 (83.1–87.7)	72.9 (70.1–75.5)	84.5 (81.8–86.8)	<0.001
Anterior wall of bladder	71.0 (69.1–72.9)	72.3 (70.2–74.3)	63.4 (53.8–71.5)	56.7 (48.8–63.8)	71.5 (62.1-78.9)	<0.001
Posterior wall of bladder	83.0 (82.2–83.8)	83.4 (82.4–84.2)	85.8 (82.0–88. 8)	70.8 (66.5–74.6)	83.0 (78.9–86.3)	<0.001
Bladder neck	74.1 (72.6–75.5)	75.3 (73.7–76.9)	73.7 (67.0–79.2)	56.2 (50.0–61.8)	73.8 (67.2–79.3)	<0.001
Ureteric orifice	90.2 (89.2–91.0)	90.40 (89.4–91.3)	88.7 (83.6–92.2)	87.4 (81.5–91.6)	84.6 (78.7–89.0)	<0.001
Urachus	53.0 (47.2–58.4)	56.3 (49.2–62.8)	52.1 (33.1–68.2)	39.6 (23.0–55.7)	44.9 (27.4–61.0)	0.192
Overlapping lesion of bladder	64.6 (63.9–65.4)	65.4 (64.6–66.2)	62.9 (59.4–66.1)	52.2 (49.1–55.1)	65.8 (61.9–69.3)	<0.001
Bladder, NOS	75.2 (74.8–75.6)	76.2 (75.8–76.6)	72.3 (70.5–74.0)	59.2 (57.5–60.9)	71.8 (69.9–73.7)	<0.001
**Treatment**						
SC	68.7 (67.8–69.6)	69.6 (68.6–70.6)	66.7 (62.5–70.6)	56.2 (52.2–60.0)	73.9 (69.7–77.7)	0.132
Chemotherapy	62.0 (60.4–63.4)	63.6 (61.9–65.2)	50.2 (42.2–57.6)	42.2 (36.2–48.0)	65.8 (58.7–72.1)	0.288
SRC	33.4 (31.6–35.2)	33.3 (31.3–35.3)	37.7 (29.6–45.8)	26.9 (21.1–33.0)	40.2 (31.1–49.0)	<0.001
Surgery	37.9 (36.6–39.2)	39.2 (37.8–40.6)	33.4 (26.3–40.6)	25.4 (21.3–29.8)	35.5 (28.7–42.3)	<0.001
SR	33.4 (32.2–34.6)	34.2 (32.9–35.5)	33.9 (27.3–40.5)	22.3 (18.7–26.2)	36.8 (30.8–42.8)	<0.001
Radiation	26.8 (25.1–28.5)	27.8 (26.0–29.7)	34.3 (24.4–44.5)	12.5 (8.5–17.0)	30.8 (22.4–39.5)	0.087
RC	19.2 (15.3–23.4)	18.9 (14.5–23.7)	26.5 (8.5–48.9)	18.6 (9.6–29.9)	13.1 (3.27–29.8)	0.184

Cancers diagnosed in the period of 1973–2008, and followed up to 12/31/2013 in the SEER 18 database. In each cell, estimated rate (95% confidence interval).

### Discussion

Bladder cancer is the most common malignancy of the urinary system. It is also one of the top ten common tumors of the body. Current studies investigated racial differences for a small number of racial groups, most of them focus on blacks and whites only^20,21^. Some other studies just cared about a small number of outcome variables^22^. In this study, UBC patients in the United States among non-Hispanic whites, Hispanic whites, blacks and APIs are compared comprehensively on the basis of SEER data. Significant racial differences are found in terms of patients’ characteristics, clinical-pathologic features, incidence and survival. Such results can be informative for cancer epidemiologists, clinicians, and policy-makers.

The observed gender, age, and tumor grade distributions are similar to those in the literature^18,21^. The statistical analyses suggest the existing of racial differences on the distributions of gender, marital status, age, age at diagnosis, survival time, histological types, grade, stage, treatment, tumor size, regional nodes negative, and tumor location. These findings are mostly consistent with those in the literature^23,24^. It is possible that the complex interactions of genetic makeup, living habits, occupational exposures, and environmental exposures cause the racial differences among UBC patients^25^.

Racial differences in incidence have been studied in a few publications^6,20^. Chen and others limited their study to blacks and whites, analyzed SEER data from 2010 to 2013, and found that blacks have lower incidence rates than whites^6^. Our analysis includes much wider racial distributions and generates more comprehensive results. Non-Hispanic whites have higher incidence rates of UBC than blacks while Hispanic whites have lower incidence rates than blacks and APIs have lower incidence rates than other races. Multiple factors may have contributions to the racial differences in incidence. One possible risk factor is smoking. As some studies reported that smoking significantly elevated bladder cancer risk (odds ratio = 2.4) and there are some known bladder carcinogens just like 2-napthylamine in tobaccos^26–28^. Non-Hispanic whites have been found that have a higher proportion of smokers compared with Hispanic whites, blacks, and Asian American^28^. The racial difference in UBC can also be attributed to occupational exposures. Numerous studies have demonstrated that workers in high-risk occupations involving exposure to dyes, rubber, leather, ink, or paints have an increased risk of bladder cancer. Three of the chemicals used in these industries (2-naphthylamine, benzidine, and 4-aminobiphenyl) are classified as human carcinogens by the International Agency for Research on Cancer (IARC)^29,30^. Some researchers already found the racial differences between non-Hispanic whites, Hispanic whites and blacks in exposure to occupational hazards^31^. Coffee drinking has been studied extensively as a potential risk factor, but the inconsistency of the observed association suggests that the relationship is quite weak^11^. In our study, we also found that when stratified by histological types, for SCC and Carcinoma, blacks have the highest incidence rate. Kantor and others also found that blacks have the highest incidence rate of SCC^32^.The high incidence of SCC appears to be related to a high incidence of endemic schistosomiasis in blacks^33^. Eradication of schistosomiasis can effectively reduce the incidence of SCC in blacks. It’s not clear why blacks have the highest incidence of Carcinoma: further studies are needed.

The racial difference for survival outcome of UBC patients has been studied in some publications. Scosyrev and others compared the mortality rates of blacks and whites adjusted for grade, stage, and cell type in 2009, and found that for a time less than three years since diagnosis, blacks have higher mortality rates than whites^18^. Hollenbeck and others used SEER data for the years from 1992 through 2002 and found that blacks have an approximately 70% greater risk of cancer-related death compared with whites^21^. In our analysis, the survival advantage of whites is observed and blacks have the worst survival rate. The results are the same as those mentioned above. When stratified by treatment strategies, racial differences still exist for SR, SRC, and surgery, with blacks having the lowest survival rate. So we think that treatment may not be the main reason for the racial difference in survival rate. Hollenbeck’s research already showed that the use of aggressive therapies (cystectomy, systemic chemotherapy, radiation) was found to be similar among black patients and white patients. After adjusting for the difference in treatment intensity and provider effects, the hazard ratios for blacks and whites just decrease 0.01^21^. Racial differences could also be observed from our study after stratified by stage and histological types, with blacks still have the worst survival rate. These findings support a recent SEER study that reported an excess hazard of death from bladder cancer among blacks despite adjusting for age, stage and grade^18^. Delays in diagnosis resulting in higher stage disease and different histological types may not be the reasons for racial difference in survival. Walker *et al*. have also show that incidence of more advanced tumors is similar among blacks and whites^20^. Genetic differences or tumors with poor prognostic molecular markers may correlate with more aggressive bladder cancer in blacks. But now, we don’t have clear evidence. Additionally, different lifestyles could also be the reason of the racial difference of UBC. Parkin *et al*. already show that survival is better in the developed areas than in developing areas^34^ because people are more likely to have unhealthy lifestyles such as smoking, physical inactivity, and consumption of calorie-dense food in less developed areas^35^. Racial differences in survival may also be the end result of disparities in the healthcare provided to different races. Blacks may receive a lower quality of care relatively, which can reflect differential treatment by race.

In this study, we also found that APIs have the highest survival rate instead of whites in some cases. For histological types NPTCC and SCC, APIs have the highest survival rate. The possible reason may be different treatment strategies for some specific histological type in different racial groups. Additionally, different genetic makeup and lifestyle behaviors may contribute to this result. For treatment strategies SC, Chemotherapy, SRC, and SR, APIs have the highest survival rate than other racial groups. It reminds us that APIs may be more suitable for these treatment strategies mentioned above than other racial groups. This result can provide a reference for choosing the appropriate treatment strategies for different racial groups. The survival advantage of APIs is observed for stage II and overlapping lesion of bladder. Reasons for that need further researches. We can also learn from our study that tumor location in urachus and overlapping lesion of bladder has a lower survival rate than other locations. Tumor locations of UBC have rarely been studied before. It can provide a reference for prevention and treatment of bladder cancer.

### Limitations

The SEER database is analyzed in this study because it is the current largest cancer registry in the United States. However, it still has many limitations. Firstly, errors may arise in tumor classification and staging in multiple sites. But we do not expect a series of systematic errors correlated with ethnicity. Secondly, data collected in SEER may not be comprehensive enough. For instance, there are no data of genetic factors or lifestyle that may contribute to UBC. Furthermore, SEER does not collect data regarding insurance type, educational level, socioeconomic status, availability to healthcare and other factors that affect one’s treatment, and as a consequence, the survival outcome. Additionally, the details about treatments are not recorded in SEER, such as surgical methods, chemotherapy dosage and so on. All above unmeasured patient differences may confound correlations between race and outcomes. Also, the study on SEER data is focus on patients from the United States only. It is not clear whether the results hold for other nations. In addition, although the sample size is at the same level as in related studies^18,36^, it would possible be over powered.

## Conclusion

This study shows that there exist significant racial differences in patient characteristics, incidence and survivals among UBC patients in the U.S. The exact cause of such difference is not revealed. The identified racial difference could be informative and leveraged by UBC epidemiologists and clinicians despite the limitations.
